# A Third Generation Water Bath Based Blackbody Source

**DOI:** 10.6028/jres.100.044

**Published:** 1995

**Authors:** Joel B. Fowler

**Affiliations:** National Institute of Standards and Technology, Gaithersburg, MD 20899-0001

**Keywords:** aperture, blackbody, cavity, conical, emissivity, radiation, radiometry, reflectance, source, temperature, thermometer, water bath

## Abstract

A third generation water bath based black-body source has been designed and constructed in the Radiometric Physics Division at the National Institute of Standards and Technology, Gaithersburg, MD. The goal of this work was to design a large aperture blackbody source with improved temporal stability and reproducibility compared with earlier designs, as well as improved ease of use. These blackbody sources operate in the 278 K to 353 K range with water temperature combined standard uncertainties of 3.5 mK to 7.8 mK. The calculated emissivity of these sources is 0.9997 with a relative standard uncertainty of 0.0003. With a 50 mm limiting aperture at the cavity; entrance, the emissivity increases to 0.99997.

## 1. Introduction

In 1985 NIST designed and built a first generation water bath based blackbody source [1] with a wide aperture cylindrical-conical cavity design. The cavity was coated on the inside with a specular black gloss enamel paint and immersed in the water medium of a commercially available temperature-controlled water bath. Although the cavity used in this first-generation source had the same large 10.8 cm diameter aperture as the one described here, it was shallower, resulting in a lower effective emissivity. The bath used in this earlier blackbody design was unstable at temperatures near ambient. In 1987 NIST designed and built a second generation water bath based on blackbody source with the same large-area aperture as the first generation, but with a 75 % deeper cavity which increased the number of reflections in the cone from three to four with a resultant increase in the effective emissivity. The new bath had an increased temperature stability resulting in a 20 mK to 50 mK combined standard uncertainty^1^ (that is, estimated standard deviation as stated in [1]), including the water temperature stability and uniformity at any of the temperatures in its operating range. The second generation bath had temperature instabilities in the temperature range from 10 °C above to 10 °C below ambient room temperature, which necessitated the use of an auxiliary cooling loop to maintain the above combined standard uncertainty in this range. The apparent source of these water temperature instabilities was the bath configuration and internal temperature control loop stability.

The third generation blackbody source described in this paper has a wide (10.8 cm) diameter aperture and an extended conical cavity section similar to that of the second generation design. The water temperature stability; of this new blackbody source is ±2 mK or less over many days; the temperature uniformity of the water volume is ±2.0 mK at the lowest temperature in it’s operating range and ±5.0 at the high end of its operating range of 278 to 353, as measured using the resistance thermometry detailed later in this paper. The increased stability is due to state of the art control loop electronics and the thermo-mechanical configuration of the bath. The increased uniformity is due primarily to the physical configuration of the bath.

## 2. Design

**Fig. 1:**
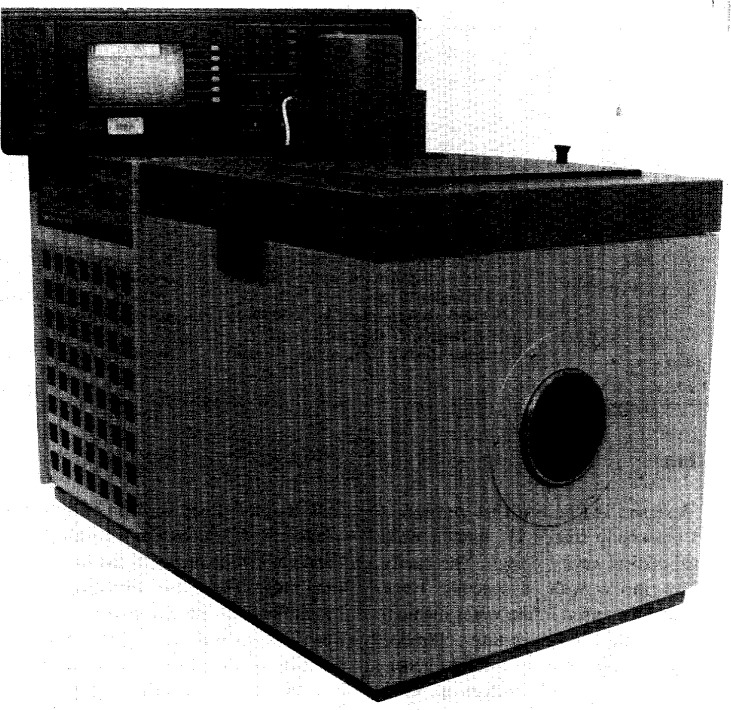
Third generation water bath based blackbody source.

This blackbody source incorporates a specially modified Hart Scientific Model 7008^2^ temperature-controlled water bath with GPIB control capability to heat and cool the water in which the cavity is immersed and a Hart Scientific electronic thermometer Model 1575 used in conjunction with a Thermometrics precision thermistor probe model ES-210 “temperature standard” to accurately measure the temperature of the water in the bath. Hereafter, the model 7008 water bath will be referred to as the bath, the model 1575 electronic thermometer as the electronic thermometer, and the model ES-210 thermistor probe as the thermistor probe unless otherwise specified. The entire apparatus is shown in Fig. 1.

The bath is well suited for this application because of the excellent temperature uniformity and stability of the water in the bath. The cooling and heating sources are laminated into a single plate covering the bottom of the bath well. The water in the bath well is agitated by a low speed double stirrer. The cavity is mounted in the side of the bath allowing deflection of the warm air from the evaporator coil of the refrigeration unit away from the cavity mouth; this reduces air currents in the vicinity of the cavity. Without the cavity installed, the bath is stable to better than ±1 for extended periods of time and the water uniformity is better than ±1 throughout the volume over the entire operating range. With the cavity installed, the instability values increase to ±2 or better over periods of days with a ±5 maximum nonuniformity at any temperature in the operating range.

### 2.1 Cavity Design and Construction

The cavity was constructed using oxygen-free copper. The conical portion was machined from a solid round bar and the cylindrical portion was machined from a section of tubing. These two parts, along with a mounting ring, were then brazed together in a vacuum oven using a high-copper-content-alloy brazing material. The oxygen-free copper has a very high thermal conductivity of 3.88 W/(cm) [2], which improves the thermal uniformity of the cavity and decreases the thermal resistance of the cavity wall. The outer surface of the cavity was plated with a thin layer of gold over nickel to retard oxidation of the copper surface.

**Fig. 2:**
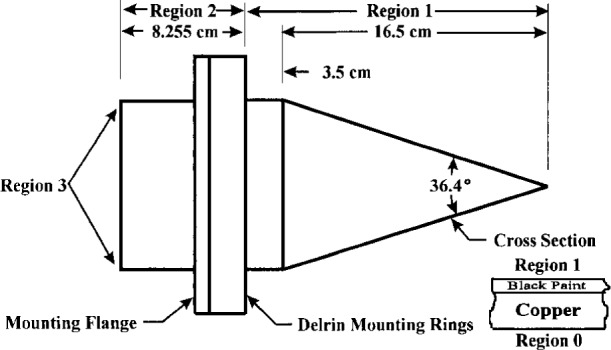
Layout of cavity details.

Enamel paint was applied to the interior surface of the cavity wall by setting the cavity in a ring stand with the tip of the cavity down and introducing approximately 1 fluid ounce (four 0.25 fluid ounce bottles) of Testor’s black model paint into the cavity. The cavity was then rotated axially while being slowly tipped from vertical to horizontal; thereby coating the entire interior surface with the paint. Once coated, the cavity was placed on a sheet of paper with the tip pointing up, thereby permitting the excess paint to drain out of the cavity while at the same time keeping the paint fluid by trapping the vapors from the paint inside the cavity. Once the excess paint had drained out of the cavity, in about 4 h, the cavity was supported approximately 12 mm above the paper and the paint permitted to harden. Support in this manner reduces the chance of distortion of the paint due to changes in the force acting on the paint due to gravity and keeps dust away from the inside cavity surface while the paint is hardening. The paint was tack free and firm after 2 days. The cavity was then baked at 50 °C for 8 h. Useful dimensions and other properties of the cavity section are shown in Fig. 2.

## 3. Control and Measurement

The bath electronics controls the heating cycle through the use of a proportional-integral-derivative (PID) control loop. The cooling cycle is preset at one of two levels depending on the cooling requirements. Cooling and heating modes are controlled from the front panel or by the computer interface. The heating and cooling circuitry allow achievement of bulk water temperature stability of better than ±2 with the cavity installed. The temperature of the water in the bath is measured with the electronic thermometer in conjunction with the thermistor probe. The electronic thermometer contributes a temperature measurement uncertainty of 1 mK and the thermistor probe contributes an additional temperature measurement uncertainty of 1.5 mK. The temperature setpoint of the bath is controlled by, and the temperature data from the electronic thermometer is read by, a digital computer utilizing a GPIB interface and software developed at NIST.

The bath may be controlled by either control panel settings or the GPIB interface. The method of setting the temperature is similar either way. When under program control, the desired temperature is input to the program. The bath is then commanded by the program to go to the desired temperature setpoint in the coarse setting mode which has a 1 K setting accuracy. The bath temperature is then read by the electronic thermometer until the temperature has stabilized close to the setpoint, with an instability of ±5 mK. The bath is then commanded in the high resolution setting mode (which is capable of setting the bath temperature relative to the measured bath temperature to better than ±1.0 mK) to change the temperature by the difference between the desired temperature and the actual measured temperature. After this final temperature setpoint adjustment, the bath will attain a temperature setpoint well within the requirements of this application. The computer program monitors and logs the temperature of the bath continuously while at this setpoint.

## 4. Temperature Measurements

### 4.1 Measurement of Water-Volume Thermal Uniformity Around the Cavity

The water surrounding the cavity wall was measured with the electronic thermometer, using two thermistor probes, one on each of the two input channels of the instrument. One thermistor probe was positioned at a fixed reference point near to the cavity tip and the other thermistor probe was movable around the perimeter of the cavity. The movable thermistor probe was positioned at ten locations, at three different levels around the outline of the cavity using a special fixture. The three levels correspond to the centerline of the cavity, bottom edge of the cavity, and top edge of the cavity. Immersion effects were minimized by encasing the area above the thermistor probes within an insulated dome. This permitted the entire volume above the thermistor probes to be engulfed by water vapor or air at nearly the same temperature as the immersed portion, thus minimizing heat conduction along the probe casing and thereby reducing the immersion loss errors to a negligible level compared to the other errors in the temperature measurement system. The resulting measured values for the water temperature uniformity are given in Table 1.

These measurements indicate that the maximum deviation of the water temperature at each of the three levels around the periphery of the cavity varies from 0.0 mK near the cavity tip to +4 mK near the bath wall adjacent to the cavity at 278 K, has no variation between the cavity tip and a position near the bath wall adjacent to the cavity at 303 K, varies from 0.0 mK near the cavity tip to −5 mK near the bath wall adjacent to the cavity at 333 K, and varies from −2 mK near the cavity tip to −7 mK near the bath wall adjacent to the cavity at 353 K referenced to the fixed probe. The highest values of the deviations of the water temperature at each of the above four temperatures were chosen as the conservative values of the water temperature nonuniformity at those temperatures.

### 4.2 Cavity Lip Temperature

A differential thermocouple thermometer was used to measure the temperature drop between the immersed cavity components and the outside cavity lip. One thermocouple was imbedded in white heat sink compound and pressed against the outer lip of the cavity with a 20 cm length of its cable in contact with the cavity lip. The other probe was covered with the same compound and pressed against the wetted portion of the conical section of the cavity while immersed in the bath water with approximately the same 20 cm cable length in the water with the sensor. Each channel of the differential thermometer was calibrated using a bath whose temperature was measured and set with the Hart thermometer to read exactly 353.00 K. A difference of 4.1 K was measured between the cavity lip and the immersed tip of the cavity. Both channels were then rechecked at the conclusion of the measurement and they still indicated 353.00 K. The standard uncertainty of the differential thermometer was 50 mK, as both indicated in the manufacturer’s data sheet and verified by the electronic thermometer. The temperature of 353.00 K was chosen as the worst case as it represents the largest deviation from ambient temperature.

### 4.3 Water Temperature Uncertainties

The water temperature uncertainties can be divided into two categories. The first is the combined contribution from the external temperature measurement system consisting of the electronic thermometer and the thermistor probe, along with the possible temperature control errors due to the water bath temperature control characteristics. The second consists of the uncertainties of the thermodynamic properties and the behavior of the cavity, the effects of the cavity interior coating, and the environmental effects due to such things as convection, stray air currents, etc.

The uncertainties associated with the control and external measurement of the temperature of the water in the bath are straightforward in that only the contributions from the water bath temperature control and the electronic thermometer-thermistor probe combination need to be considered. The bath control circuitry contributes an absolute temperature setting uncertainty of 1 K without external temperature measurement and less than 1.0 mK with external temperature measurement when using the high-resolution-setting mode as stated by the manufacturer. The external temperature measurement uncertainty is due to the thermistor probe and the electronic thermometer. The thermistor probe contributes 1.5 mK uncertainty as calibrated at the factory and referenced to a standard traceable to NIST. The Hart electronic thermometer contributes an uncertainty of 1 mK or less as calibrated by the factory and traceable to a NIST standard, resulting in a combined standard uncertainty of 1.8 mK for the combination of the electronic thermometer and the thermistor probe. The uncertainty in the bath temperature stability is due to the bath control loop which has an instability of less than ±2 mK and has no contribution from the external thermometry for a given setpoint temperature within the operating range of the instrument.

Uniformity of the bath temperature, as discussed in Sec. 4.1, is typically ±1 mK without the cavity inserted. Upon insertion of the cavity, this value degrades slightly to ±2 mK for the range 78 K to 313 K and ±5 mK for the range 313 K to 353 K. Table 2 shows the uncertainties associated with the temperature of the water in the bath.

The combined standard uncertainty of the bath water temperature may be calculated by adding in quadrature the standard uncertainties associated with the water temperature nonuniformity, bath temperature setting error, possible thermistor probe immersion error, temperature measurement error due to the thermistor probe, and temperature measurement error due to the electronic thermometer, yielding a standard uncertainty of 5.3 mK at 278 K, a standard uncertainty of 3.5 mK at 303 K, a standard uncertainty of 6.1 mK at 333 K, and a standard uncertainty of 7.8 mK at 353 K as shown in Table 1 and Table 2. These are worst case standard uncertainties of the water temperature at any point in the bath surrounding the cavity, but does not take into account the interface between the water and the cavity and the thermodynamics of the cavity. These last two factors are taken into account in the calculations described later in this paper.

These blackbody sources will generally be used with a lower cost Hart Scientific Model 1506 electronic thermometer with a temperature measurement standard uncertainty of 6 mK, as stated by the manufacturer and referenced to a NIST standard when used in combination with the thermistor probe (same type used with the model 1575 thermometer). The substitution of the model 1506 thermometer will increase the combined standard uncertainty of the water temperature measurement to 9.8 mK, which is near the maximum 10 mK standard uncertainty required for this application. The more expensive model 1575 thermometer was used in these tests only to determine how stable and accurate this blackbody source could be with high accuracy components.

## 5. Cavity Emissivity

Ideally, when the walls of the cavity are in local thermodynamic equilibrium, the emissivity of the cavity *ϵ* is 1 minus the cavity reflectance. Using this fact, we may calculate an approximation for the emissivity *ϵ* based on the assumption that the reflectance of the interior wall of the cavity is the sum of a perfectly specular component *ρ*_s_ and a perfectly diffuse component *ρ*_d_. The specular reflectance is divided into two components: one to account for the specular reflectance at normal incidence and one to account for the specular reflectance at lower angles of incidence. This is necessary, as the angle of incidence of each reflection after entering the cavity varies. For radiation entering the cavity at near normal incidence, the emissivity *ϵ* is expressed by [3]

$$\epsilon =1-\left({\rho_{\text{ss}}}^{2}{\rho_{\text{sh}}}^{2}\right)-dF_{13}\rho_{d}$$ (1)

where d*F*_13_ is the differential configuration factor which describes the fraction of the radiation emitted from a differential area d*A* on the cavity wall which exits through the opening of the cavity, *ρ*_sh_ is the specular reflectance of the cavity surface at high angles of incidence for the first two reflections, and *ρ*_ss_ is the specular reflectance of the cavity surface at smaller angles of incidence for the remaining two reflections. We have ignored the small variation of *ϵ* with wavelength *λ*. Four reflections inside the cavity were chosen due to physical limitations of the bath and the manufacture of the cavity and diminishing returns from additional reflections. These four reflections equate to an absorption of 99.998 % of the radiation entering the cavity. Equation (1), though not a worst case approximation, may be used for a worst case analysis by choosing conservative estimates for *ρ*_d_, *ρ*_sh_, and *ρ*_ss_.

The measured reflectance of a witness sample of the black gloss coating used inside the cavity but applied to the same copper material from which the cavity was machined is 5 % total reflectance up to 10 µm, 7 % up to 20 µm and rises rapidly past 20 µm at near normal incidence. The reflectance value is increased from 5 % to 10 % for the larger angles of incidence. Though no data were available for the diffuse reflectance beyond 2.5 µm, the diffuse reflectance is known to be <0.2 % between 800 nm and 2.5 µm and become less diffuse and more specular with increasing wavelength. The reflectance for this cavity was chosen to be a conservative value of *ρ*_sh_ = 10 % specular for the larger angles of incidence *ρ*_ss_ = 5 % specular for the smaller angles of incidence, and *ρ*_d_ = 0.2 % for the diffuse reflectance. The value of the differential configuration factor d*F*_13_ was calculated for nominal cavity dimensions and varies from 0.03 near the tip of the conical section of the cavity to 0.07 near the cylindrical-conical intersection. A conservative value of d*F*_13_ = 0.07 was chosen.

Utilizing the above values, Eq. (1) yields 0.9997 for a lower bound for the emissivity *ϵ* with 0.9997±0.0003 for a conservative estimate for the spectral range of 1 µm to 30 µm. If a 50 mm diameter aperture with high infrared reflectance on the side facing the cavity were added to the front of the cylindrical portion of the cavity and the calculation repeated, the emissivity increases to near 0.99997 at normal incidence to the cavity.

As a check of the above calculations, the emissivity was recalculated utilizing a computer program written by Prokhorov and Sapritsky [3] for the calculation of blackbody emissivity. For the same parameters as used in the above calculations, this computer program yields an effective emissivity of 0.9998 at normal incidence. Recalculating with the addition of a 50 mm aperture in front of the cylindrical portion of the cavity, the emissivity normal to the cavity increased to 0.99996. This program also can account for nonuniform temperature distributions over the cross sectional and longitudinal dimensions of the cavity. Randomly varying the temperature uniformity input to the computer program by as much as ±100 mK, a much worse case than our 5 mK maximum measured nonuniformity, the normal emissivity calculated was never less than 0.9991 without the 50 mm aperture or less than 0.99991 with the 50 mm aperture. This confirms our assumption that the nonuniformity of the surface temperature of the cavity as measured in this instrument is not significant.

Effects such as air currents and the consequences of off-axis viewing have been ignored and will be addressed in a report on the radiometric testing of the blackbody currently being performed at NIST.

### 5.1 Temperature Distribution Over the Interior Cavity Surface

The worst case approximations used to estimate the temperature drops in regions 1 and 2, shown in Fig. 2, for this the cavity are as follows:

1. Region 3 is at a uniform temperature throughout (*T*_3_).
2. Region 2 is at a uniform temperature throughout (*T*_2_).
3. The worst case value for the temperature in region 2 is the temperature at the very edge of the cavity lip.
4. Region 0, the surface in contact with the bath water, is at a uniform temperature *T*_0_ which is the same temperature as the bath water.

For high accuracy measurements, the temperature of the bath water must be very stable and accurately measured. The water in this bath was accurately measured for stability and absolute temperature as outlined in Sec. 4.1 and meets this requirement in excess of the extent necessary to achieve the desired quality of the source. The term quality will be described later.

### 5.2 Temperature Drop Across the Cavity Wall and the Black Paint

The differential heat conduction across the cavity wall in region 1 and radiating out of the cavity is given by:

$$dP=\left(T_{0}-T_{1}\right)/\left(d_{\text{cu}}/K_{\text{cu}}+d_{\text{bp}}/K_{\text{bp}}\right),$$ (2)

where *d*_cu_ is the cavity wall thickness, *K*_cu_ is the thermal conductivity for the copper wall of the cavity, *d*_bp_ is the thickness of the paint, and *K*_bp_ is the thermal conductivity of the enamel paint. For the assumed thermal equilibrium, the above quantity must balance the net differential radiant power leaving the surface of the paint on the inside of the cavity wall at any point in region 1 as shown in Fig. 2. This quantity d*P* is given by

$$dP=dF_{13}\sigma \left(T_{1}^{4}-T_{3}^{4}\right)+\left(dF_{12}-dF_{13}\right)\sigma \left(T_{1}^{4}-T_{2}^{4}\right)$$ (3)

where *σ* is the Stefan-Bolzmann constant, and d*F_ij_* is the differential configuration factor from the point of interest in region *i* to all of region *j*. Because the temperature difference between *T*_0_ and *T*_1_ is small, the error introduced by approximating *T*_1_^4^ by

$$T_{1}^{4}=T_{0}^{4}+T_{0}^{3}\Delta T$$ (4)

is negligible, where Δ*T* = *T*_1_−*T*_0_ is the temperature drop across the cavity wall and paint. Equations (3) and (4) may be solved simultaneously for Δ*T* in closed form

$$\Delta T=\frac{-\beta T_{0}\left(dF_{13}[1-{\left(T_{3}/T_{0}\right)}^{4}]+\left[dF_{12}-dF_{13}\right]\left[1-{\left(T_{2}/T_{0}\right)}^{4}\right]\right)}{1+4dF_{12}\beta },$$ (5)

where

$$\beta =\sigma T_{0}^{3}(\left(d_{\text{cu}}/K_{\text{cu}}+d_{\text{bp}}/K_{\text{bp}}\right).$$ (6)

Table 3 enumerates the nominal values used in the evaluation of Δ*T* in Region 3. The above analysis is similar to the analysis presented in NBS Technical Note 1228 [1] and has been modified to reflect the changes in the design of the new blackbody design.

The paint thickness was measured by taking the difference between the thickness of the coated metal blank used for the witness sample in the measurement of the reflectance of the black paint before and after coating. Although the method of coating the sample was performed to closely approximate the inside of the cavity, the estimate of 0.005 cm may be incorrect by up to 50 %, therefore *d*_bp_ = (0.005 ± 0.0025) cm has been chosen as a conservative estimate.

Values for the temperature drop Δ*T* across the cavity wall at the intersection of the conical and cylindrical portions of the cavity which are totally immersed in the temperature controlled water were calculated and are shown in Table 4 for several water temperatures, along with the associated uncertainties.

## 6. Blackbody Quality

The blackbody quality accounts for the effects of temperature gradients between the water in the bath and the cavity surface, and the cavity wall reflectance in a single quantity [4,5]. Quality is defined here in terms of a reference temperature, which is conveniently the temperature which is actually being measured during the operation of the blackbody, the water temperature in this case. It is the ratio of two radiances that are important: the actual cavity radiance, and the ideal Planck-law radiance at this reference temperature.

A simple expression for the quality of a blackbody of this type can therefore be expressed [1] by

$$Q=\epsilon \left[\exp\left(C_{2}/\lambda T_{0}\right)-1\right]/\left[\exp\left(C_{2}/\lambda T\right)-1\right]$$ (7)

where *ϵ* is the emissivity (again ignoring the small variations of *ϵ* with *λ*), *λ* is the wavelength of interest, *T* is the effective cavity temperature, *T*_0_ is the reference temperature and *C*_2_ is the the second radiation constant. *Q* is simply the calculated emissivity modified by the ratio of the ideal and actual Planck law radiances.

Taking the first two terms of a Taylor series expansion of the right hand side of Eq. (7) and substituting Δ*T = T*−*T*_0_ when the second term is small compared to unity, Eq. (7) may be approximated [1] by

$$Q\approx \epsilon \left[1+\left(\Delta T/T_{0}\right)\left(C_{2}/\lambda T_{0}\right)/[1-\exp\left(-C_{2}/\Delta T_{0}\right)\right].$$ (8)

### 6.1 Overall Blackbody Quality

We can use Eq. (8) to calculate the quality of the blackbody at any wavelength and to calculate the uncertainty of the quality using the values calculated for Δ*T* and *ϵ*. An equation for the uncertainty in the blackbody quality is [1]

$$u_{c,r}\left(Q\right)=\sqrt{Q\left[{\left(u\left(\epsilon \right)/\epsilon \right)}^{2}+{\left(F\left(C_{2}/\lambda T_{0}\right)u\left(T\right)/T_{0}\right)}^{2}\right]},$$ (9)

where

F(x)=x/[1−exp(−x)]. (10)

may be derived from Eq. (7).

**Fig. 3:**
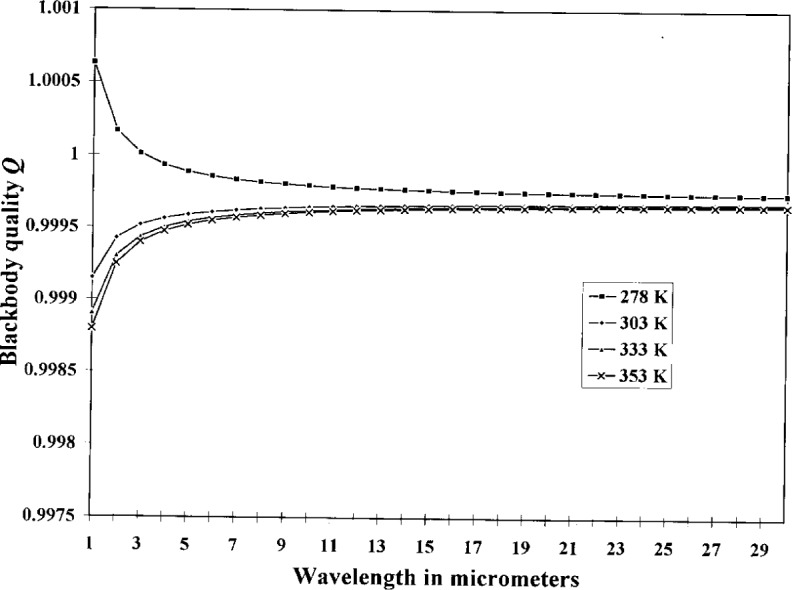
Blackbody quality.

**Fig. 4:**
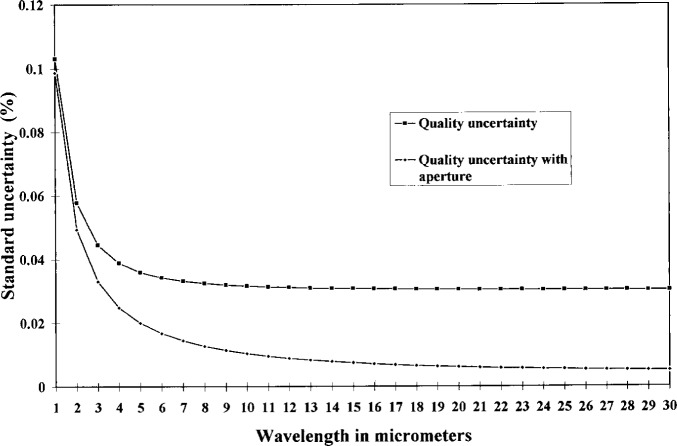
Blackbody quality uncertainty.

Table 5 shows the relevant values used in the calculation of the quality and the uncertainty of the quality, and Figs. 3 and 4 graph the quality and it’s uncertainty versus wavelength.

## 7. Conclusion

A high quality thermometer was used in the evaluation and in the operation of the water bath during testing. In the normal use of this instrument, a thermometer only slightly better than the expected performance need be used. The Hart Scientific Model 1506 electronic thermometer with a thermistor probe suits this need very nicely. Substitution of the Model 1506 only degrades the performance by the increased uncertainty of the thermometer. The temperature measurement combined standard uncertainty of the Model 1506 electronic thermometer when used in conjunction with the thermistor probe is 6.2 mK. The blackbody quality would only decrease by 0.1 % at long wavelengths and 0.01 % at the shorter wavelengths if used with the lower accuracy thermometer.

The uniformity and stability of this new generation water-bath-based blackbody shows definite improvement over past designs, both in ease of use and overall quality. The design exceeds our goal of 10 mK combined standard uncertainty of the water temperature, whether used with the Hart Super Thermometer or the Model 1506 Metrology thermometer. The calculated emissivity is very high and we expect excellent radiometric characteristics. Radiometric measurements are currently being conducted at NIST and will be the subject of a future paper.

## Figures and Tables

**Table 1:** Water temperature nonuniformity and standard uncertainty values used in the uncertainty analysis at four set-point temperatures

Water temperature (K)	Nonuniformity (mK)	Standard uncertainty value used (mK)
278	−1 to +4	4
303	−1 to 0	0
333	0 to −5	5
353	0 to −7	7

**Table 2:** Water temperature measurement and control errors and standard uncertainties used in uncertainty analysis

Source of uncertainty	Range of possible temperature error values (mK)	Standard uncertainty (mK)
Electronic thermometer	±1.0	1.0
Thermistor probe	±1.5	1.5
Stability of bath	±2.0	2.0
Bath setting	±1.0	1.0
Est. immersion loss	±2.0	2.0

**Table 3:** Values of parameters used to calculate Δ*T* from Eq. (5)

Parameter	Value
Cavity internal diameter	10.7 cm
Length of cylindrical cavity section	10.9 cm
Full angle of cavity	38°
Thickness of cavity wall	0.4 cm
Thickness of black paint	0.005 cm
Thermal conductivity of cavity wall	3.8 W/(cm K)
Thermal conductivity of black paint	0.0018 W/(cm K)

**Table 4:** Calculated temperature drop across the cavity wall from the bath water to the inside cavity wall (ambient temperature 298 K)

Water temperature (K)	Temperature drop (mK)	Standard uncertainty (mK)
278	+0.9	0.9
283	+0.5	0.5
293	0	0
303	−0.5	0.5
313	−1.1	1.1
323	−1.7	1.7
333	−2.4	2.4
343	−3.2	3.2
353	−4.0	4.0
363	−4.9	4.9

**Table 5:** Parameters used in the calculation of the blackbody quality and uncertainty using Eq. (1) and Eqs. (8) and (9).

Δ*T* (mK)	Standard uncertainty *u*(Δ*T*)^a^ (mK)	Restrictions (K)
+0.9	5.3	*T*_0_ = 278
+0.5	4.9	*T*_0_ = 283
0.0	3.7	*T*_0_ = 293
−0.5	3.5	*T*_0_ = 303
−1.1	3.8	*T*_0_ = 313
−1.7	4.9	*T*_0_ = 323
−2.4	6.1	*T*_0_ = 333
−3.2	7.0	*T*_0_ = 343
−4.0	7.3	*T*_0_ = 353
−4.9	7.8	*T*_0_ = 363

*ϵ*	Standard uncertainty *u*(Δ*ϵ*)	Restrictions

0.9997	0.0003	No aperture
0.99997	0.00003	50 mm aperture

a These values are only valid when viewing the conical section of the cavity.

## Footnotes

1 All uncertainties given in this paper are standard uncertainties (i.e., 1 standard deviation estimates) unless otherwise specified.

2 Certain commercial equipment, instruments, or materials are identified in this paper to foster understanding. Such identification does not imply recommendation or endorsement by the National Institute of Standards and Technology, nor does it imply that the materials or equipment identified are necessarily the best available for the purpose.
